# The paradox of the artificial intelligence system development process: the use case of corporate wellness programs using smart wearables

**DOI:** 10.1007/s00146-022-01562-4

**Published:** 2022-09-26

**Authors:** Alessandra Angelucci, Ziyue Li, Niya Stoimenova, Stefano Canali

**Affiliations:** 1 Dipartimento di Elettronica, Informazione e Bioingegneria, Politecnico di Milano, Milan, Italy; 2 The Cologne Institute of Information Systems, Faculty of Management, Economics and Social Sciences, University of Cologne, Cologne, Germany; 3 Department of Industrial Engineering and Decision Analytics, The Hong Kong University of Science and Technology, Hong Kong, China; 4 Department of Industrial Design, Delft University of Technology, Delft, Netherlands; 5 META—Social Sciences and Humanities for Science and Technology, Politecnico di Milano, Milan, Italy

**Keywords:** Artificial intelligence, Fairness, Classification model, Corporate wellness program, Smartwatches

## Abstract

Artificial intelligence (AI) systems have been widely applied to various contexts, including high-stake decision processes in healthcare, banking, and judicial systems. Some developed AI models fail to offer a fair output for specific minority groups, sparking comprehensive discussions about AI fairness. We argue that the development of AI systems is marked by a central paradox: the less participation one stakeholder has within the AI system’s life cycle, the more influence they have over the way the system will function. This means that the impact on the fairness of the system is in the hands of those who are less impacted by it. However, most of the existing works ignore how different aspects of AI fairness are dynamically and adaptively affected by different stages of AI system development. To this end, we present a use case to discuss fairness in the development of corporate wellness programs using smart wearables and AI algorithms to analyze data. The four key stakeholders throughout this type of AI system development process are presented. These stakeholders are called service designer, algorithm designer, system deployer, and end-user. We identify three core aspects of AI fairness, namely, contextual fairness, model fairness, and device fairness. We propose a relative contribution of the four stakeholders to the three aspects of fairness. Furthermore, we propose the boundaries and interactions between the four roles, from which we make our conclusion about the possible unfairness in such an AI developing process.

## Introduction

An increasing number of decisions regarding the daily lives of human beings are being controlled by Artificial Intelligence (AI) algorithms in spheres ranging from healthcare, transportation, and education to college admissions, recruitment, provision of loans, and many more realms. AI models have been developed and deployed in various applications with satisfactory performance and exponentially increasing popularity: for example, natural language process for text semantic analysis (Gabrilovich and Markovitch 2007), regression models for disease risk prediction (Goff et al. 2014), classification models for hiring decisions (Black and van Esch 2020) and criminal identifications (Tayal et al. 2015), face recognition for emotion understanding (Balconi et al. 2011), human activity recognition for the remote monitoring of the activity level of patients (Angelucci et al. 2020a), and so on. AI systems are becoming deeply embedded into the fabric of society and, consequently, have an increasing influence on a wide range of decisions made by humans every day.

Since AI systems now touch on many aspects of our lives, it is crucial to develop AI algorithms that are not only accurate but also unbiased and fair in performing their tasks, such as monitoring, prediction, and recommendations (Pessach and Shmueli 2020). Decisions made by AI systems often lead to perpetuating harmful biases and result in discrimination based on gender, race, or sexual preferences (Rahwan et al. 2019). The notion of fairness is largely debated and discussed from different viewpoints, and different ideas and approaches are proposed in the literature and adopted in practice. A common ground that has been agreed upon is that a fair AI model should provide the same accurate result for the underrepresented minority population as the majority population, regardless of sensitive attributes such as gender, race, and sexual orientation (Kim et al. 2019; Pfohl et al. 2019). While different aspects of fairness have been discussed extensively, these analyses have often come from either a philosophical and ethical background or a computer science and engineering background only. This has led to a polarization towards specific aspects of fairness depending on the background of the scholars studying the issue and to poor adoption of guidelines and ethical codes by computer scientists and engineers in their practical activities (Hagendorff 2020). In this paper, we bring together work from philosophy, ethics, computer science, and engineering and argue that the development of AI systems is marked by a central paradox: the less participation one stakeholder has within the AI system’s life cycle, the more influence they have over the way the system will function. With the term ‘life cycle’, we define all the steps from the time the AI solution is conceived and designed to the actual use of the solution; the life cycle includes the deployment and validation of the solution. This means that the impact on the fairness of the system is in the hands of those who are less impacted by it.

To illustrate this paradox, we discuss a specific application of AI systems in a concrete context: corporate wellness programs using smart wearables and AI to analyze data. We approach this as a ‘use case’. The term comes from software and systems engineering, where it is used to discuss scenarios for the use of a piece of software. In our work, we use this term to frame our analysis of the application of AI to corporate wellness programs with smart wearables—we do not refer to an individual type of AI, program, or wearable, but rather discuss elements and features across different applications. The use of AI in these programs is increasing, and there is a critical literature looking at the ethical and social limits of these attempts (Ajunwa 2020b). On this basis, in our analysis we distinguish the different roles played by service designers, algorithm designers, system deployers, and end-users in developing and using fair AI systems. We focus on the ways in which fairness needs to be ensured while developing AI systems in this context and identify and discuss three aspects of fairness—contextual fairness, model fairness, and device fairness. On this basis, we discuss the impact of different stakeholders on the different aspects of fairness in AI systems, identifying and discussing a central paradox.

Our research in this article is based on a literature review of current philosophical discussions on the ethics of AI and fairness, automated decision-making and the workplace, stakeholder management and technological development. We are a heterogeneous group of early-career scholars with diverse expertise in biomedical engineering, computer and data science, responsible AI, philosophy, and ethics. Our experience at different stages of the development and assessment of AI systems gives us a diverse perspective of the common practices and pitfalls of the AI development process and helps us approach the issue of AI systems’ fairness with an eye on both conceptual and technical considerations. Finally, our specific expertise on wearables comes of use in analyzing technical solutions that are being or could be used in the use case of corporate wellness programs.

The structure of the paper is as follows. In Sect. 2, we discuss the use case of corporate wellness programs and present the different roles involved in AI system development. In Sect. 3, we explain the three aspects of fairness, i.e., contextual fairness, model fairness, and device fairness. Section 4 presents the interaction between the stakeholders and the different aspects of fairness as they are seen in the different roles.

## Use case: corporate wellness programs using smart wearables

We focus on a use case to exemplify and elaborate upon the challenges that arise during the different stages of development of a specific AI system: corporate wellness programs using smart wearables and AI techniques to analyze the data. Our focus is influenced and inspired by recent technological, societal, and public developments around the use of similar solutions. For example, in the field of telemedicine, where smart wearables are being increasingly employed for several applications (Angelucci and Aliverti 2020), among which pre-symptomatic COVID-19 detection (Mishra et al. 2020). Smart wearables can collect data with very high sampling rates (e.g., some devices sample their sensors at 10 Hz, meaning that ten samples per second can be available) in a continuous fashion. So-called big data are quickly obtained by using these devices (Park et al. 2014), so AI methods are the primary choice to analyze such outputs and provide feedback to the users. Furthermore, AI methods can be based on machine learning or deep learning; the “learning” implies that the more data are collected, the better the accuracy becomes, making such techniques ideal for programs involving a constantly increasing number of available data. This is true for instance in the case of human activity recognition, which is performed by several wearables with AI algorithms taking as input data from motion sensors; such algorithms are becoming more and more performing because more data are being collected over time and classification can become more accurate every time the devices are updated with the new embedded algorithms. Another example of an embedded algorithm is the possibility of detecting pathological conditions from the signals collected by smart wearables, such as the notorious atrial fibrillation detection algorithm embedded in the Apple Watch (Perez et al. 2019).

Generally, the term “wellness program” describes any program designed to promote health or prevent disease. Ever since the introduction of smart wearables, they have been improving healthcare and providing personalized health advice, lauded as the primary uses of the technology (Farr 2020). Big tech companies such as Apple and Google are working in close collaboration with hospitals, clinicians, health coaches, private insurances (e.g., the collaboration between Apple and Aetna (Shieber 2019)) and governments (e.g., collaborations between the Singaporean government and Fitbit (Somauroo 2019) and then with Apple (Farr 2020).^1^ In general, workplace wellness programs have experienced a resurgence within the last decade: healthier workers mean fewer sick days and lower healthcare costs for a firm (Ajunwa et al. 2016).

Despite these promises and potential benefits, several risks and burdens are connected to AI systems for wellness programs. For instance, depending on the read parameters, algorithms could be able to discover which and how many female employees might be pregnant. This might be positive on the one hand because the woman would be nudged to visit a gynecologist soon, but it could also cause her not to be promoted or fairly evaluated. The data collected by wellness programs may reveal employees that are likely to represent higher healthcare costs for the employer (Ajunwa 2020a), therefore another possible discrimination could consist in systematically firing “costly” employees (Roberts 2013). On this basis, Ajunwa and colleagues (Ajunwa et al. 2016) have argued that an ethically grounded wellness program should maintain an impenetrable barrier between the information it collects and the employer. Furthermore, any information shared with the employer should be in the form of aggregated statistics and should be anonymized to prevent the individual employee from being targeted for discrimination. Moreover, the collection of data from wellness programs, while not always physically invasive, holds potential for privacy invasions that, ethically, workers should be informed of (Ajunwa et al. 2016). Wellness programs collect significant amounts of personal health information (PHI) from the employees. Such PHI represents lucrative data. This information may be sold to pharmaceutical companies interested in developing drugs or to data brokers to be used in creating various types of lists. Thus, an important part of an ethically grounded workplace wellness program is transparency concerning data collection, storage, and ownership. Another issue is the accuracy of the data being collected by wearables. Research on the functioning of wearables indicates that not all devices have the same quality, and many commercially available wearables might present errors or inaccuracies (Bayoumy et al. 2021). Finally, while most of these programs worldwide are voluntary, some scholars have expressed some concern about the incentives and penalties tied to these programs. Past research has focused on the use of incentives which may be characterized as carrots (rewards) or sticks (penalties), and which could take the form of modified premiums, smaller copays or deductibles, cash, gift cards, or merchandise (Cawley 2014).

These considerations are the starting point for our use case, which we study with a primary focus on fairness. Let us consider how a company board could offer their employees a new smart wearable to monitor their wellbeing utilizing AI algorithms. The purpose of this wellness program could be to encourage the employees to stay healthy both in terms of performing physical activity and monitoring physiological parameters. Once the wellness program is designed and deployed, employees could choose whether to take part in the initiative and, if they do, start wearing the wearable and receiving feedback after their data are processed with AI algorithms. Examples of data that can be acquired with a wearable are electrocardiogram (ECG), heart rate, peripheral blood oxygen saturation, respiratory rate, motion data, and calories consumption. Once data are acquired, they can be processed with AI algorithms, which are particularly suitable for the large amount of data that are made available. For instance, motion data can be processed to obtain human activity recognition and estimations of the activity level of a person, thus determining if the subject has a sedentary or active lifestyle. Other parameters can be used to detect diseases: the Apple Watch, for example, is certified to detect atrial fibrillation (AF) from its ECG sensors (Perez et al. 2019). Heart rate (Kinnunen et al. 2020) and respiratory rate (Angelucci et al. 2020b) can be measured during exercise to determine if a person is experiencing some distress.

During the design, deployment, and usage of a corporate wellness program, different stakeholders are involved. There are several definitions of possible roles in the AI workflow in the literature (Meske et al. 2021). Following results of this literature, in this case we can distinguish at least four roles:

1. **Service designer** who translates the requirements of the specific social-technical system to the functioning of the AI system to be developed.
2. **Algorithm designer** who develops new AI algorithms from scratch, thus advancing the knowledge in the field of AI itself.
3. **System deployer** who deploys AI models to use them for specific uses using existing algorithms.
4. **End-user** who experiences a simple input/output relationship without any knowledge of the model.

The focus of the **service designer** is to ensure that a concept for an AI system can be designed in such a way that, once integrated within the broader socio-technical system, its behavior will trigger the intended use and outcomes. In our use case, the service designers are those who decide how to implement the corporate wellness program: they choose what data to use as inputs, what outputs the AI-based system should give and how, the type of device that should be used (e.g., smartwatches), and all these details. The service designer decides the specifications of the corporate wellness program and knows how the population of employees is composed. For instance, service designers can decide whether the program is only “passively” monitoring parameters (e.g., determining if a person’s lifestyle is active or not) or also “actively” encouraging to be more active if needed. The process to design a service is iterative (Zomerdijk and Voss 2010): to achieve the desired results, the service designer first identifies the purpose of the potential solution. This is usually achieved through an iterative process of data collection (both qualitative and quantitative) that comes from an overview of available technical and commercial solutions. An initial version of the AI system is prototyped and is then tested with a subsample of the intended users, usually on volunteers or the very members of the development team. Such early prototype testing not only allows to falsify the existing hypotheses about how the solution should behave and what outcomes it produces, but it also allows to identify potential unintended (both negative and positive) consequences. The resulting insights become the starting point for the next iteration of updating the solution’s design so that it can mitigate the negative consequences that were identified and amplify the positive ones. In the end, the outcome of this process serves as a guideline for the system deployer on how the AI model should behave so that its use and outcomes would not deviate from the ones intended by the designer.

The role of the **algorithm designer** is to design and program new AI models from scratch. Algorithm design involves computer science work such as quite a bit of algorithm theory and research. Algorithm designers are not usually directly employed in companies, unless such companies are specialized in AI solutions, but are rather academicians or industry researchers in the field of AI. Despite their apparent distance from the specifics of the use case, their design choices influence the performances and outcomes of the models that thousands of deployers around the world will use. Several models exist, and the number of available models is likely to expand in the following years due to the very high worldwide interest in the field of AI. In the present use case, the algorithm designer does not interact with the service designer or the system deployer, even though they are the person who knows most of the mathematical and technical characteristics of the AI models that will be used.

**System deployers** are usually engineers hired by a company to deal with a specific solution. In this use case, the system deployers are likely biomedical engineers or computer scientists with a healthcare specialization. It is crucial to distinguish between algorithm designers and system deployers, which are strictly linked to the fact that the number of scientists using existing AI solutions is increasing, even if most of them are not experts in the field of AI itself. The deployer has the role to utilize existing models to create a solution and insert that into a contextual process. This phase is crucial since the AI solution is used to make inferences “in the wild”, meaning that it should be effective and robust while working with data that can be drastically different from the one used in its training and its initial testing during the development stage. Furthermore, in this stage, the system should be able to be generalized, meaning that it should prove its robustness and validity on any dataset, not just the one that has been used to deploy the solution. In the development of corporate wellness programs, the service designer explains the intended input and output of the AI system; the role of the system deployer is to choose the suitable devices, collect the data, and train and fine-tune the AI models that will provide the given outputs. The system deployer has knowledge of the use case and interacts with the service designer in the prototyping phase.

Algorithm designers differ from system deployers because designers contribute to advancing knowledge in the field of AI, for instance, by creating novel neural network models, while deployers fine-tune existing networks, e.g., by using previously deployed libraries such as Python’s *keras* (Ketkar 2017) or *TensorFlow* (Abadi et al. 2016). Designers and deployers may coincide in rare cases, but they generally have different levels of expertise in the field of AI itself. Deployers tend to have more domain knowledge and know the specifics of the single-use cases in which AI is used.

The **end-users** are the company employees who take part in the corporate wellness program, regularly use the wearables, and receive outputs coming from the AI model. They may have no knowledge of what is going on in the system; therefore, the outputs of the AI model could be biased and unfair without even being noticed. The end-users base their decisions on the outputs of the AI system even though their understanding of the working principle is minimal, if not none.

## Aspects of fairness involved in the AI system implementation

The use case we have discussed so far in the paper shows a context of use of an AI system which, from an ethical point of view, needs to be fair. As a concept and feature of technological solutions, fairness has been widely discussed in different disciplines and areas of the literature, where it is often defined and used in different ways. Issues that have been discussed as problems of fairness include for instance issues of discrimination and mistreatment at the individual level, negative experiences of advantages and disadvantages for specific social groups, as well as moral harms related to justice and equity, and quantitative and mathematical issues in allocation and representation according to certain criteria (Smith 2020). Within this context, fairness has more specific connotations and raises more specific issues as part of the debate on AI and machine learning, in connection to several and infamous cases where AI and machine learning systems were found to discriminate against specific individuals and thus treating them unfairly with respect to other individuals, for example in the justice system (Massaro et al. 2022). As a consequence, an extensive literature in philosophy, ethics, sociology, etc. has analysed issues of fairness arising from the increasing use of AI systems and worked on both conceptual frameworks and technical solutions (e.g., Binns 2018; Floridi and Cowls 2019; Tsamados et al. 2022). At the same time, for industry and big tech companies, the focus on ensuring an AI system is and remains fair is mostly based on improving the fairness strictly from the algorithmic and mathematical point of view, without much attention to other features and the more general ethical implications of fairness (Smith 2020). Building on this literature, for the purpose of our work in this article we start with the basic idea of fairness as the idea of ‘treating like cases alike’(van Nood and Yeomans 2021) and the connotation of this approach in the health context, where the literature of fairness has framed the concept as a way of identifying and discussing a just conduct of individuals when they interact with health services and thus a way of ensuring that people are not treated in unjust ways because of bias, discrimination, lack of consideration (Olsen 2011).

On this basis, we see the present use case as a situation where issues of fairness may arise and the fairness of several aspects of the system need to be ensured. Following results from the philosophical literature on the need of AI fairness to be contextual and the issues of one-size-fits-all approaches (Abu-Elyounes 2020), we develop a multi-faceted analysis of fairness in this context and we argue that we need to distinguish the following three aspects of fairness at least:

1. **Model fairness**, i.e., the aspect of fairness that enables us to prevent unlawful discrimination (e.g., discrimination against the protected attributes such as age, gender, and race) from the algorithmic and mathematical point of view.
2. **Contextual fairness**, i.e., the aspect of fairness where the factors that influence what is considered fair are highly dependent on the socio-technical system in which the AI-powered solution is to be implemented.
3. **Device fairness**, i.e., the aspect of fairness related to sensors, technical specifications, and general means by which data are collected.

### Model fairness

Race, nation, and gender bias are commonly observed in the AI models, and the examples in literature are countless. A disease risk prediction model has been found to overestimate risk for female patients, Chinese patients, or globally, as well as also underestimate risk for other groups such as Korean women (Pfohl et al. 2019). An AI-empowered recruiting algorithm was shown to prefer male candidates and penalize resumés that included the word “women” (Bornstein 2018). In the last years, the problem of fairness emerged so clearly that toolboxes to ensure fairness have been developed, an example of which is the Python toolkit for algorithmic fairness “AI Fairness 360”, developed in 2018 (Bellamy et al. 2019). The package includes a comprehensive set of fairness metrics for datasets and models, explanations for these metrics, and algorithms to mitigate bias in datasets and models. To guide the AI models in the direction of advocating more fairness, algorithm and model fairness have been explored significantly in the past several years, with as many as 21 different definitions of “model fairness” developed (Caton and Haas 2020; Kleinberg et al. 2018; Narayanan 2018; Pessach and Shmueli 2020; Verma and Rubin 2018).

**Fig. 1:**
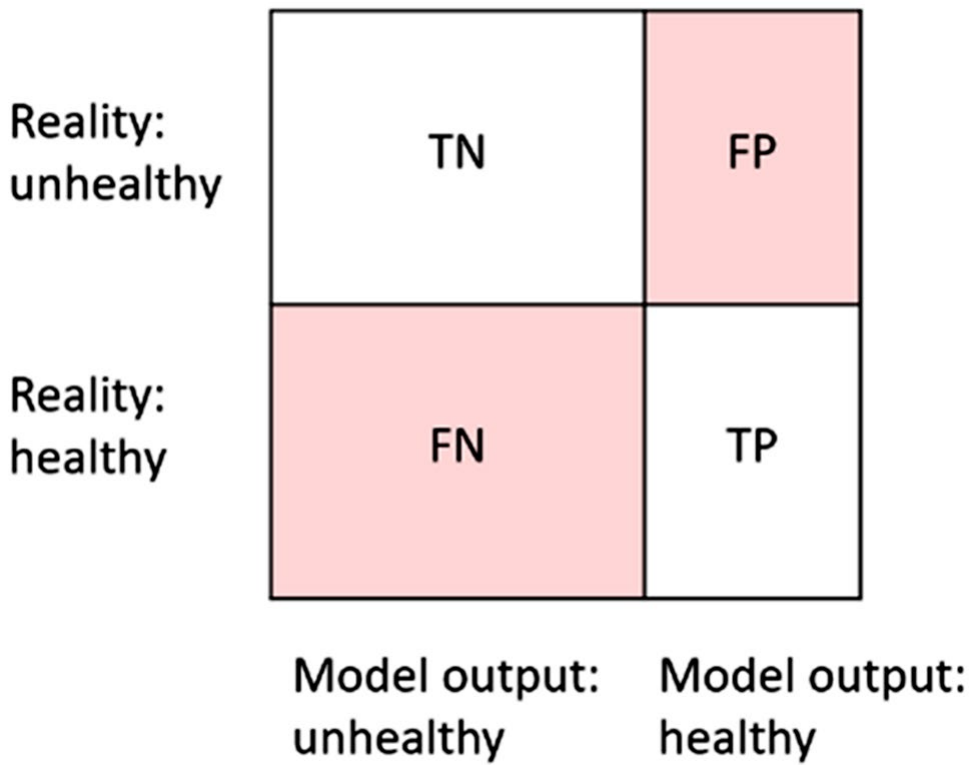
Model outputs compared to reality: there are true negatives (TN), false negatives (FN), false positives (FP), true positives (TP)

Scholars generally agree is that a fair AI model should ideally provide the same accurate result for the underrepresented minority population as the majority population, regardless of sensitive attributes such as gender, race, and sexual orientation (Kim et al. 2019; Pfohl et al. 2019). Even in the single perspective of model fairness only, however, different stakeholders pursue the model fairness with different focus or preferences; this applies to the use case presented in this paper, too. Figure 1 shows a simple table with the possible model outputs compared to the reality in a generalized classification problem: there are true negatives (TN), false negatives (FN), false positives (FP), and true positives (TP). In the case of corporate wellness programs, a true negative is someone who is actually unhealthy and is correctly classified as such, whereas a true positive is someone who is truly healthy and in this case, classification is also correct. False negatives and false positives are those who are not correctly classified.

In our use case, once there is the decision to implement a corporate wellness program, a service designer is appointed and must provide general guidelines for the technical team that will develop the solution. The major concerns of the service designers while they give instructions to collect the data to train the AI model are if the selected dataset is demographically balanced and if the obtained AI model does not discriminate with respect to protected attributes such as race, gender, or sexual orientation. Some parameters of interest are precision and recall: precision quantifies how many positive results are true positives, while recall quantifies how many true positives are correctly classified as such. In the present use case, precision quantifies how many employees are healthy among those labeled as “healthy” employees, while recall quantifies how many healthy employees are correctly classified as healthy. The technical team that will implement this solution is particularly concerned with these parameters, and its goal is to maximize the performance of the model to achieve the best possible results. For the employees that will individually use the devices as end-users, it is particularly important for them to know the possibility that they are incorrectly classified as “healthy” when they are “unhealthy” or vice-versa.

No single metric captures all the desirable properties of a model, and several metrics are typically reported to summarize a model’s performance. Generally, these measures are not easily understandable by people without technical knowledge of AI. Focusing on precision or recall depends on the application and what type of outcome is desired, as is explained in the following section dedicated to contextual fairness.

### Contextual fairness

**Fig. 2:**
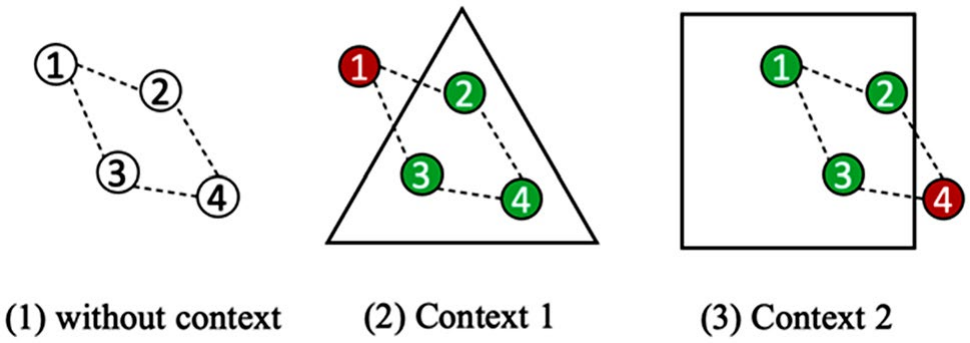
Fairness with respect to context

As reported in the previous sub-section, several researchers agree that fairness depends on the context (Abu-Elyounes 2020). Figure 2 explains fairness with respect to context: each circle with a number is a ‘node’ (i.e., connected point) of a system, and the context is the shape in which a system is contained. As shown in Fig. 2, it is subjective, if not impossible, to determine whether one of the shown nodes is fair or unfair: node 1 is regarded as unfair in context 1, whereas in context 4, node 1 is fair, and node 4 instead is regarded as unfair. A practical example is the inclusion or exclusion of given subjects from a health or wellbeing program: if a program is targeted at children, then excluding adults is not unfair but simply dictated by the context of the program.

The solutions to this problem that have been put forward are predominantly centered around introducing metrics that provide mathematical formulations that can quantify the degree of fairness or bias in an AI system (Foulds and Pan 2020). Even though all mathematical formulations are derived from a notion of egalitarianism, which means that all parameters (e.g., precision, recall) are considered equally important from an algorithmic standpoint (Lee et al. 2021), fairness is not only an algorithmic concept but also a societal, highly contextually dependent one (Lee et al. 2021; Zhang et al. 2020). A risk prediction instrument that is fair with respect to mathematical fairness criteria may still result in undesired consequences for one of its stakeholders depending on how and where it is used (Chouldechova 2017). Therefore, AI systems have to be seen not only as technical artifacts, but also as social constructions that incorporate diverse viewpoints (Brandao et al. 2020; Selbst et al. 2019; Zhang et al. 2020). In this use case, the wellness program is at a corporate level, so the composition of the population in terms of gender, age, and other parameters is a priori known and cannot be ignored while deploying a solution. The final solution must be tailored to the needs of the specific social group that will ultimately use it.

Little guidance exists on how to incorporate the socio-technical requirements of fairness into the functional behavior of the AI model. However, Selbst et al. (2019) put forward five guidelines AI service designers should consider:

1. the entire socio-technical system should be modeled by understanding the social context and its stakeholders and defining what fairness means for them.
2. the solution and the AI model powering it are context-dependent. Therefore, before attempting to transfer these to another context, one should gauge to what extent doing so will be fair.
3. social context must dictate the choice of the mathematical formula of fairness.
4. try to anticipate the potential unintended consequences of the interaction between the AI system and the larger socio-technical system.
5. be aware that AI might not be the solution to the problem at hand.

In the present use case, we think that contextual fairness should consider the final purpose of the initiative. The aim of the company is to encourage its employees to be more active and healthier by using wearables. Suppose a possible criticality is detected, such as out-of-range physiological parameters or too little physical activity, arguably, the system should behave as “strictly” as possible, which means that the AI models should be able to detect potentially harmful situations easily and warn the user, at the cost of causing a false alarm. However, in the field of healthcare and wellbeing, there is a large debate on the topic: a false positive (i.e., a healthy person that is classified as unhealthy by the algorithm) might cause unwanted alarm, but a false negative (i.e., an unhealthy person that is classified as healthy) might lead to a late diagnosis.^2^

A crucial aspect consists in a correct education of the employees, who should be aware that the AI systems unavoidably make mistakes from time to time, even if deployed to the best of the available knowledge. Another critical aspect is that model deployers may not have sufficient background to understand how solutions should be designed for a specific purpose, especially in the fields where complex ethical issues arise, such as healthcare. This might lead to further errors in addition to the unavoidable ones. In this use case, encouraging and nudging the employees to be more active is preferred, but it is also important to ensure that the specificities of the company are considered. If the company has a largely young population, for instance, it must be considered that activity-related recommendations for older employees might differ. Also, it is important to inform the employees whether the employed devices and AI solutions have medical-grade certification; for instance, the Apple Watch proprietary algorithm is certified for atrial fibrillation detection via its ECG sensor (Perez et al. 2019) but not for other conditions. Obtaining a medical certification is a long and complex process in developed countries. Therefore, it is extremely unlikely that a company autonomously deploys certified AI models. AI regulators and entities responsible for the certification of such systems are not taken into consideration in this paper due to the excessive differences among international regulations.

### Device fairness

In terms of fairness, there are aspects of fairness related to sensors, technical specifications, general means by which data are collected. In many existing situations, non-AI solutions are already discriminatory, and introducing AI runs the risk of simply perpetuating and replicating these flaws. An example can be people coming from socio-economically deprived environments, who are likely to have less health literacy and so might not be able to interpret the output given by health monitoring devices, with or without AI. It has been discussed in the literature how a generalized use of AI can be fair and inclusive with some categories, for instance, people with disabilities (Trewin et al. 2019). The literature is often focused on the context and/or the model, especially in the field of computer science, but the technical characteristics of the chosen device are often overlooked. It is, however, crucial to know the specifications of the employed wearables, even if the companies producing them do not share complete nor detailed datasheets. Also, both service designers and deployers must be able to distinguish between devices that have obtained medical-grade certifications and those that did not, especially in the case of a corporate wellness program that is meant to provide health information to the users.

The same technology may not be perceived the same way by all its users. In fact, there are people with different digital literacy, and this might generate unfairness, for instance, towards the elderly or people with disabilities (cognitive or physical). This might lead to lower adherence to wellness programs using AI-based systems, with the consequence that even fewer data will be collected, and such groups tend to be even less represented. This applies to this use case: minorities among the employees might be de facto excluded from the program, with the double effect of biasing the AI system and renouncing the possibility of being part of a wellness program that is intended to improve their health and wellbeing.

Not only is the choice of technology a possible factor of unfairness, but the technology itself might have different performances in different groups. It is known that there are specific measurement systems that might be affected by the characteristics of the subject, such as skin color. For instance, a concern with the PPG (photoplethysmography) technology for detecting pulse rate, which is widely used in smartwatches and wearables to estimate heart rate and peripheral blood oxygen saturation, is related to the melanin concentration and pigmentation of the skin. Melanin is known to be highly absorbent to light, and thereby the measurement can be attenuated and lead to errors in measurements. This was demonstrated in previous studies (Fallow et al. 2013); therefore, it is necessary to choose a producer that has already taken into account this aspect while developing the device that is used to acquire the data. Otherwise, there may be an intrinsic bias in the system even before applying AI. This is one of the issues that should be first analyzed by the system deployer.

## Interactions between the four roles: a central paradox in AI development 

Each development stage narrows down the scope of the potential AI system. For instance, the service designer decides on the functional behavior the AI system should exhibit, as well as the intended way it should be used, thus having a very strong influence on contextual fairness. The algorithm designer further narrows down the scope of the system by defining the parameters and performance of the new model that could achieve a given purpose, which is usually explicitly defined when the model is shared with the public. Finally, the system deployer must work within the confines of the scope initially set by the service designer and then narrowed down by the algorithm designer. Thus, they must integrate and embed the model in a specific context so that it could deliver value to its stakeholders but remain within the previously set boundaries.

On the other hand, the time spent on each stage of the AI system lifecycle is disproportionate to the level of influence each role has on the boundaries within which the system needs to operate. Although service designers set the overall boundaries, they participate only at the very beginning of the system’s life cycle. The algorithm designer spends time on developing the algorithm, which usually takes significantly longer than the conceptual design stage but is generally a “once and for all” work since a new algorithm can be used by thousands of system deployers around the world. Further still, the role most involved during the entire lifecycle of the AI system is the system deployer. However, this role is also the one who has the least amount of influence over the scope within which the system should operate. Finally, the end-users are the ones that are most involved during the entire lifecycle, and their behavior influences the model itself due to its self-learning properties. However, the model remains a black box to the end-users, and they are neither privy to its working mechanism nor can they consciously influence the way the system will behave.

Our analysis of this use case and the roles played by different stakeholders leads us to argue that the development of AI systems is marked by a central paradox: the less participation one stakeholder has within the AI system’s life cycle, the more influence they have over the way the system will function. Specifically, the service designer knows the context very well but generally has no technical knowledge on how to deploy the technology, and the system deployer writes the code based on the specifications declared by the service designer (specific to the service) and the algorithm designer (specific to the model).

In the discussed use case, the service designers, i.e., the members of the team that implements the program, decide the scope and the extent of the wellness program. The system deployer is generally an engineer hired for the task which only follows the instructions coming from above and makes choices based on what is declared in the software documentation by the algorithm designer, who has a much deeper knowledge of the mathematics behind the system. The final end-user receives information about his or her health and fitness status and recommendations on how to be healthier yet generally has no understanding of what lies behind the solution being used. This paradox makes the AI development process potentially unfair since a system might fail to consider what the end-user experiences and desires from the systems, and the end-user is left with no power at all.

The three aspects of fairness are considered during the four stages of AI development, but with different levels of effects to contribute and make a difference, as shown in Table 1. The proposed scale goes from 0, which equals to no contribution, to 3, which represents a fundamental contribution, and is a qualitative scale based on our knowledge of the involvement of different roles.

**Table 1:** Contributions to fairness from different stages

Four stakeholders	Specific role	Impacted aspects of fairness		
		Contextual	Model	Device
Service designer	To translate the requirements of the socio-technical system to the AI system	***	*	*
Algorithm designer	To design new AI algorithms or new methods	–	***	*
System deployer	To deploy and fine-tune AI models to use them in specific use cases	–	*	***
End-user	To simply use the input/output relationship without any knowledge of the model	–	–	–

The service designer has a general role in translating the requirements of the socio-technical system to the AI system with consideration of different specific contexts. Therefore, the most significant influence from a service designer is towards contextual fairness. Although the service designer does not know the specific model that will be used nor the devices, the defined contextual fairness by the service designer sets the boundaries for the model and device, where the following roles such as algorithm designer and system deployer need to work within. Besides, the service designer also offers the initial guidance for model fairness and device fairness. In this case, the service designer decides whether the aim of the corporate wellness program is only to monitor the status of the subject or also to predict the insurgence of pathological conditions; based on the defined requirements, the chosen model and device will be different. Therefore, to this extent, the service designer also has a moderate influence on these two types of fairness.

The algorithm designer designs and develops a machine or deep learning model or algorithm without any knowledge of the context in which the algorithm will be applied. They undoubtedly contribute to and affect the model fairness, but their contribution to contextual fairness is none. The performance of this designed model will then further affect the following deploying and fine-tuning steps. Therefore, algorithm designers can have a moderate influence on device fairness, depending on the clarity and completeness of the instructions they put in the documentation. The algorithm designer has no case-specific role since the development of novel algorithms happens independently from the final application.

The system deployer does the fine-tuning of the model to obtain a case-specific solution that is strictly related to the device(s) employed. For example, when the AI model is embedded into a PPG-sensor equipped device, as happens in the case of smartwatches, the system deployer should work with the awareness that PPG sensors generally work better on lighter skin and try to address the issue. Therefore, a system deployer contributes most to the device fairness but relatively little to context fairness, which has already been defined from upper-stream stages. The system deployer has some influences on the model fairness itself, given that they are directly involved in the deployment of the solution and can thus evaluate different possible algorithms, discuss the possible choices with the service designer, and ultimately choose the tuning parameters. After the service designers establish what the corporate wellness program should do, it is the system deployer’s role to realize it, for instance, choosing an adequate algorithm for a given purpose (e.g., convolutional neural networks are a possible choice for human activity recognition).

Finally, the end-user has a limited sociological and technical knowledge on the matter; therefore, he/she has quite a limited impact on the whole AI system and almost no influence on the three aspects of fairness, thus leading to the paradoxical situation described in the previous sections. The end-user does not have access to the information and processing behind the AI system and experiences the system as a black-box. The end-user, in this case, is the employee, who only sees the final output, for instance feedback on his or her activity level or a warning if some physiological parameter is out of range. As we have seen, wrong feedback on the health status of a person has potential negative effects, so a correct education of the employees on the potentialities and limitations of such systems is necessary to allow proper use of such technology. Even in cases in which the user’s opinion is investigated, the limited knowledge of the user on processes and algorithms prevents him or her from effectively contributing to the overall fairness of the system.

## Conclusions

In this paper, we presented the use case of corporate wellness programs carried out by means of smartwatches and AI-based data analysis. For this implementation, we have focused on contextual, model, and device fairness. Then, we have described four roles in AI development: service designer, algorithm designer, system deployer, and end-user. Starting from this use case, we have discussed the fairness of the AI systems’ development process and the notion that the process presents an intrinsic paradox, i.e., the less participation one has within the AI system’s life cycle, the more influence he/she has over the way the system will function. This central paradox and our presentation of the different steps and stages leading to it are significant contributions for the literature on AI and fairness, as we expand and specify various discussion on disproportion of power and agency between large technological companies and individual users. As a result of our analysis, we show that this disproportion is not only based on different financial or technological power but is also crucially connected to the methodologies and ways in which AI systems are developed in concrete contexts. As we have argued throughout the paper, the end user has little to no agency in the development and deployment of the technological solution and this can raise additional ethical concerns for fairness. A potential solution to these problems might consist in clearly explaining to the end-users on the conceptual basics of AI and obtaining their informed consent before they adopt the solution, i.e., before they take part in the corporate wellness program. In many cases, an end user might take important decisions without knowing why they are receiving recommendations. An additional contribution to the literature and discussion on these issues is thus that access to the technology and its outputs is not enough to gain benefits from the actual use of the technology—access to the heuristic and conceptual tool to understand the complexity and limitation of the technology is crucial.

Even though we focused on the specific use case of corporate wellness programs in this paper, many concepts could be further generalized to other applications, especially, but not exclusively, in the field of health and well-being. Many of the aspects we have discussed can be applied to individual well-being programs, not only to corporate ones. In the medical field more broadly, the aspect of contextual fairness of diagnosis must be evaluated even more specifically before the implementation and clinical usage of a device. Both a false positive and a false negative might cause damages, for instance, causing stress and anxiety to the individual user and unnecessary use of health resources.

Furthermore, it is necessary to acknowledge that developments in the field of AI, both in fundamental research and applications to consumer-facing systems, are carried out in the industry. Therefore, even if the theoretical process is as fair as possible, special attention needs to be paid to the practical application of the fairness of the development process. Given the fields of application of AI, it is only natural that most of the basic research on the topic takes place in industry rather than academia, thus making it even more complex to ensure that AI systems comply with fairness requirements.

## Data Availability

Our manuscript has no associated data.

## Footnotes

1 Apple even opened medical clinics for its employees (Farr, 2020).

2 The issue of overdiagnosis has been discussed in the literature: AI algorithms collect so much data that they might find “silent” or asymptomatic conditions just because parameters deviate from the expected ones, thus leading to overdiagnosis (Vogt et al. 2019).
